# Extra-curricular physical activity and socioeconomic status in Italian adolescents

**DOI:** 10.1186/1471-2458-6-22

**Published:** 2006-01-31

**Authors:** Giuseppe La Torre, Daniele Masala, Elisabetta De Vito, Elisa Langiano, Giovanni Capelli, Walter Ricciardi, PHASES (PHysical Activity and Socio-Economic Status) collaborative group

**Affiliations:** 1 Institute of Hygiene, Catholic University Rome, Italy.; 2 Faculty of Science of Human Movement, University of Cassino, Italy.; 3 Chair of Hygiene, University of Cassino, Italy.

## Abstract

**Background:**

The relationship between physical activity and health status has been thoroughly investigated in several studies, while the relation between physical activity and socio-economic status (SES) is less investigated. The aim of this study was to measure the extra-curricular physical activity of adolescents related to the socio-economic status (SES) of their families.

**Methods:**

The survey was carried out by submitting an anonymous questionnaire to junior high school students in the following Regions: Lazio, Abruzzo, Molise, Campania, Puglia, during the school year 2002–2003. Extra-curriculum physical activity was evaluated considering whether or not present and hours of activity weekly conducted. 2411 students agreed to participate in the study.

**Results:**

Participants were 1121 males (46.5%) and 1290 females (53.5%), aged between 11 and 17 years (median age: 12 years). 71.1% of the students reported to practice extra-curricular physical activity. Parents' educational levels and work activities play an important role in predicting students' physical activity, with the more remunerative activities and higher educational levels being more predictive.

**Conclusion:**

The results confirm the relationship between adolescents' physical activity and their families' SES. In particular, a positive relationship between participation in extra-curricular physical activity and their families high SES was found.

These data will be useful for school administrators and for politicians in order to reduce the gap between adolescents from the least and most disadvantaged families.

## Background

The relationship between physical activity and health status has been described extensively in several studies. Active lifestyles are often associated with better health status and quality of life [1-8], however, the relationship between socio-economic status (SES) and physical activity has been investigated less [9]. In regards to this subject, the definition for SES seems to be extremely variable. Giles-Corti et al., evaluates SES according to residence in low, middle or high income geographical areas [10]; Lindstrom et al., considers employment exclusively [11]; while Gordon-Larsen et al., distinguishes between the different socio-economic levels based on family income, for example, low (up to $26200), middle (between $26200 and $50000) and high (> $50000) [12]. Anyway, most of the studies on socio-economic determinants influencing physical activity choices consider education as a discriminating factor [13-16].

In Italy there isn't an individual SES classification available, territorial data (ISTAT data) is based on personal economic consumption rather than income and education. Another possible classification of personal income comes from the Italian financial acts and the different levels of income tax rates. However, this data is not easily inferable from statistical sampling including adolescents.

The aim of the present study was to investigate the relationship between adolescents' extra-curricular physical activity and the SES of their families.

## Methods

### Setting and sample size

The survey was carried out by submitting an anonymous questionnaire junior high school students randomly selected in the following regions: Lazio, Abruzzo, Molise, Campania, Puglia, during the school year 2002–2003. Sample size calculations (alpha = 0.05; beta = 0.20) suggested to sample 2502 students. We obtained informed consent for interviewing minors in our study from their parents. Students absent during the first submission were given the questionnaire in the days following.

2411 students (96.4 % of the eligible individuals) agreed to participate in the study. The research was conducted according to the Helsinki Declaration.

### Questionnaire

The questionnaire, already validated in a pilot study [17,18], contained information about the following areas:

- scholastic physical activity

- extra-curricular physical activity

- physical activity attitudes

- lifestyle habits

- parents' physical activity, education and work activity

- students' socio-demographic data

As far as concerns physical activity of the adolescents the questionnaire investigated whether or not extra-curricular physical activity is conducted, which type of physical activity is chosen by, how many hours per week are devoted to this activity.

Concerning attitudes toward physical activity, it was asked if physical activity is considered useful in preventing obesity, in socialising, and in character- building.

Moreover, we collected information on the following lifesyle habits, as dichotomous variables (yes/no): cigarette smoking, coffee and alcohol drinking.

The following data from students' parents were collected: weekly physical activity, described as intense (more than two times per week), regular (two times per week), scarce (once per week) and abstent; educational levels, indicating the level reached: Degree, Senior High school, Junior High school, Primary school; regarding work activities we used a classification cited in another study [18]: Managers/Professionals, Office workers/Skilled workers, Non-skilled workers, Unemployed, Pensioners. In this classification housewives were considered equivalent to Non-skilled workers.

### Socio-economic status

**Figure 1:**
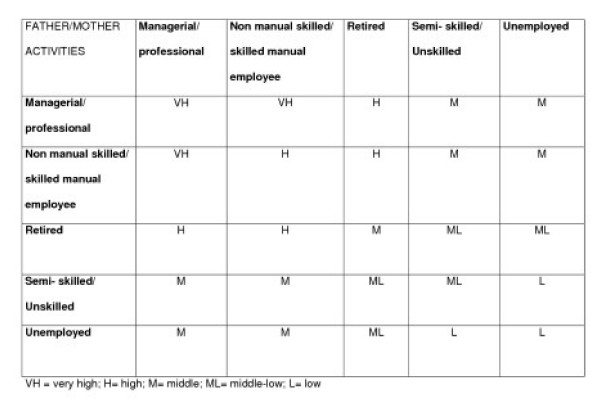
Family income index derived from the activities of the parents.

Certain information about family income was impossible to obtain from the students, therefore, SES was estimated considering parents' educational levels and work activities. A socio-economic family index, derived from the combination of parents' work activities, was calculated, using the matrix described in Fig. 1. The families' socio-economic levels were classified as: Very high, High, Middle, Middle-Low and Low.

### Statistical analysis

Data from the questionnaires was collected in a suitable relational database and analysed with SPSS statistical package.

Chi-square (χ^2^) and chi-square for trend test for qualitative variables and Student t-test for quantitative variables were applied to test the differences between the groups, considering p < 0.05 as statistically significant.

Furthermore, multiple logistic regression analysis was performed, using two different models. The first verified the influence on the variable "extra-curricular physical activity" of the following covariates: age group (reference group: 10–12 years), sex (reference group: males), educational level of both parents (reference group: primary school), father's job (reference group: unemployed), parents' physical activity (reference group: no physical activity), student's opinions about sport as a means of socialisation, character-building and prevention of obesity (reference group: negative responses).

The second regression analysis model evaluated the influence of the same covariates used in the first model (with the exception of the father's job) and that of socio-economic level (reference group: low socio-economic level) on the variable "extra-curricular physical activity". Analysis was performed using the Hosmer and Lemeshow method, with a p < 0.10 level for the insertion in the regression model of covariates considered for univariate analysis.

## Results

Participants were 1121 males (46.5%) and 1290 females (53.5%), aged between 11 and 17 years (median age 12 years). 33.7% of the students attended the first class, 38.5% the second and 27.8% the third one.

86.4% of them participated in school-based physical activity for two hours per week and 13.6% for three hours per week or more.

The types of school-based physical activity undertaken were as follows: 71.1% did gymnastics; 81.7% volleyball; 37.8% basketball; 10.7% football; 36.8% handball; 22.3% long jump; 21.6% high jump and 48.8% running.

71.1% of students declared to participate in extra-curricular physical activity with the following breakdown: 20.2% for one-two hours per week, 23.8% for three-four hours per week, while 56% stated they did five hours or more per week.

Concerning extra-curricular sports, 30.1% of the students played football, 19.7% did dancing/aerobics/gymnastics, 14.5% swam, 14.2% played volleyball, 5.8% played basket ball, 5.4% did fitness/body-building, 3.9% did martial arts, and 1.8% cycled.

370 students (15.3%) state to take nutritional supplementation before or after physical activity. 68.2% of students practising extra-curricular physical activity stated that they paid a monthly fee.

Concerning attitudes toward physical activity, 79.9% of participants considered constant physical activity useful in preventing obesity, 5.6% did not and 14.5% were unsure. 82.7% of students considered physical activity important in socialising, 5.9% did not and 11.6% remained unsure. 56.1% of responders considered physical activity important in character- building, 15.8% did not and 19.1% did not know.

As for unhealthy lifestyles, 37.8% stated that they were smokers or had started smoking, 43.8% drank coffee and 43.4% drank alcohol, especially beer, wine, alcoholic lemon drinks and vodka.

Table 1 shows parents' characteristics (physical activity levels, educational levels and work activities).

With regards to parental physical activity levels, fathers were significantly more active than mothers (χ^2 ^= 15.41; p = 0.0015). However, there were not significant differences between parental education (χ^2 ^= 4.113; p = 0.249).

The main parental work activities were: Office workers/Skilled workers (36.5%) and Non-skilled workers (34.3%) for fathers and Housewives (59.3%) and Office workers (28.3%) for mothers. According to our SES classification adolescents' families were categorised in the following way: very high 16.8%, high 14.5%, middle 37.2%, middle-low 30.1% and low 1.4%.

**Figure 2:**
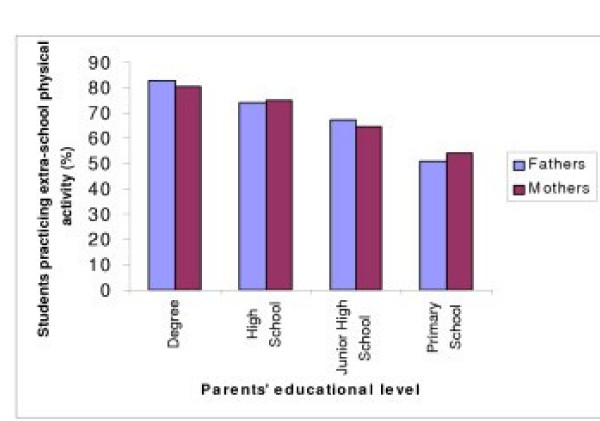
Relation between students' extra-school physical activity and parents' educational level.

**Figure 3:**
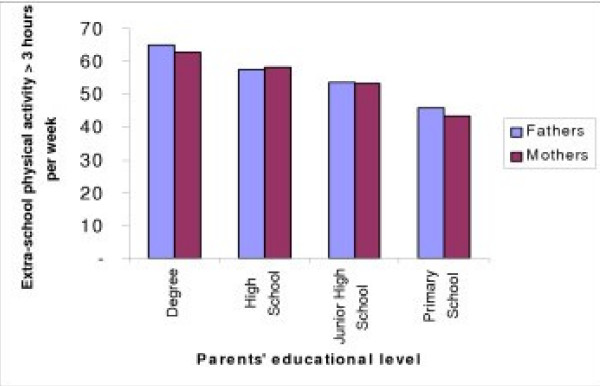
Relation between students' hours of extra-school physical activity and parents' educational level.

In relation to the SES of the students' family, the variables parents educational levels and work activities were considered. The educational level attained by the father seems to considerably influence the student's physical activity. Students with fathers who graduated with a Degree practice extra-curricular physical activity more frequently (χ^2 ^= 64.764; p < 0.0001) (Fig. 2) and more intensively (χ^2 ^= 21.091; p < 0.0001) (Fig. 3) than those with fathers characterised by lower educational attainment.

86.3% of students with fathers graduated with a Degree considered constant physical activity helpful in preventing obesity, compared to 85.2%, 80.7% and 69.4%, of students respectively, with fathers holding a diploma, a secondary-school leaving certificate or a primary school-leaving certificate (χ^2 ^= 35.421; p < 0.0001). 86.7% of students with fathers graduated with a Degree considered constant physical activity helpful in socialising, versus 84.5%, 88% and 75.5% respectively, of students with fathers holding a diploma, a secondary school-leaving certificate, a primary school/leaving certificate (χ^2 ^= 11.155; p = 0.084).

Mother's educational level also seemed to considerably influence the determinants of the student's physical activity (Fig. 2) and the total number of extra-curricular weekly physical activity hours (Fig. 3). Students with mothers graduated with a Degree were more likely to practice extra-curricular physical activity (χ^2 ^= 55.512; p < 0.0001) and undertook physical activity, for more than three hours per week (χ^2 ^= 19.65; p < 0.0001), than those whose mothers have a lower educational level.

86.4% of students with mothers graduated with a Degree and 86.5% of those whose mothers have a diploma considered physical activity important in socialising, compared to 84.6% and 78.1%, respectively, of students whose mothers have a secondary-school leaving or a primary school-leaving certificate (χ^2 ^= 12.817; p = 0.046).

83.5% of students with graduated mothers considered constant physical activity useful in preventing obesity, compared to 85.3%, 80.4% and 77%, respectively, of students with mothers holding a diploma, a secondary-school leaving certificate, a primary school-leaving certificate (χ^2 ^= 15.11; p = 0.019).

**Figure 4:**
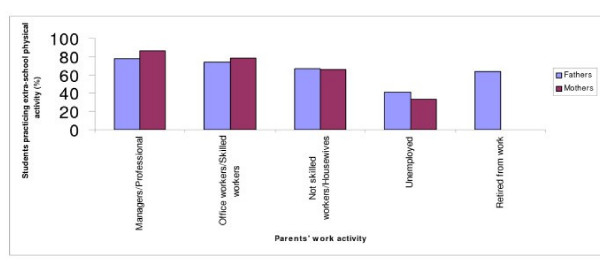
Relation between students' extra-school physical activity and parents' work activity.

Furthermore, extra-curricular physical activity is related to the parents' work activity (Fig. 4). Students whose fathers are Managers/Professional or Office-workers/Skilled workers showed a significantly higher level of extra-curricular physical activity than students with fathers who were Non-Skilled workers, Unemployed or Retired from work (χ^2 ^= 39.029; p < 0.0001).

The mother's work activity also considerably influences the students' extra-curricular physical activity (χ^2 ^= 64.319; p < 0.0001). Adolescents with mothers who are Non-skilled workers/Housewives or Unemployed undertake less extra-curricular physical activity than those with mothers who were Managers/Professionals or Office-workers/Skilled workers (Fig. 4).

**Figure 5:**
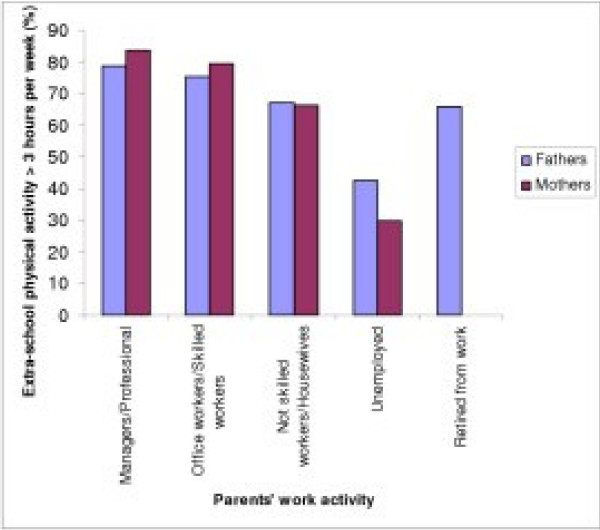
Relation between students' hours of extra-school physical activity and parents' work activity.

Weekly hours of extra-curricular physical activity show a similar trend (Fig. 5): percentages of students practising physical activity for three hours per week or more). Frequency of extra-curricular physical activity performed during the week appears to be related to the work activity level of both parents, with a statistically significant difference for fathers (χ^2 ^= 8.229; p = 0.048) and even more so for the mothers (χ^2 ^= 28.321; p < 0.0001).

Table 2 shows results of multiple logistic regression. The first model shows an association between extra-curricular physical activity and: 1) father's educational level (direct relation), 2) parents' physical activity (OR = 1.58; 95% CI: 1.24 – 2.02 for father's and OR = 1.33; 95% CI: 1.04 – 1.71 for mother's physical activity), 3) father's work activity (with a statistically significant difference between children of Managers/Professionals and Office workers/Skilled workers and children whose fathers were Unemployed), 4) attitude towards physical activity (OR = 2.25; 95% CI:1.72 – 2.94 for adolescents who considered sport important in socialising as opposed to those who did not). The likelihood of practising extra-curricular physical activity is almost 30% higher for females than it is for males.

The second model, which considers socio-economic family index, confirms the results of the first one. Moreover it shows an association between extra-curricular physical activity and SES (with ORs ranging from 1.63 and 1.96 for the indexes of High to Very high levels).

Similar results were found considering in the logistic regression models the outcome "percentages of students practising physical activity for three hours per week or more" (data not shown).

## Discussion

Several studies show evidence of a relationship between socio-economic level and health status. As demonstrated by Lowry et al. [6], higher family income is associated with lower alcohol and cigarette consumption and a lower level of sedentary behaviour. In Anglo-Saxon countries slums represent an obstacle to the practise of physical activity for several reasons ranging from absence of pavements, public parks and gardens to excessive traffic [10].

The likelihood of undertaking adequate physical activity is low for subjects who have a low family income [19] and for not skilled Workers or Unemployed [11].

Studies on the relationship between physical activity and parents' educational level [14,16,20-23,25,26] show that higher educational levels are determinant factors for children's physical activity. In particular, Sallis and coll. showed that demographic variables are good predictors of children's physical activity [21], and ethnic and SES differences are potential correlates of physical activity among adolescents [19]. In a previous study [26], in 1357 students in Central Italy, we found a strong relationship between weekly physical activity of adolescents, their BMI levels (overweight and obesity) and parents' educational levels and nutritional status, with a higher participation in physical activity shown above all in children with highly educated parents.

Results of our study confirm the relationship between family socio-economic, parents' educational levels and physical activity of adolescents.

Moreover the classification of socio-economic index used in this survey, derived by the combination of the parents' work activities (Very High, High, Middle, Middle-Low and Low), appears to be comparable to the distribution of family income levels as reported in the Italian Financial Acts (law 27/12/2002 n°289 is the latest law in chronological order) [27]. In particular the Financial Act assigns a IRPEF tax rate to every income level: tax free until 7.800,00 euro (low income); 23% for 7.800,00 to 15.000,00 euro (middle-low income); 29% for 15.000,00 to 29.000,00 euro (middle income); 31% for 29.000,00 to 32.600,00 euro (middle income); 39% for 32.600,00 to 70.000,00 (middle-high income) and 45% for greater than 70.000,00 euro (very high income).

Given these results, which are in agreement with other international surveys conducted in USA [28], South Africa [29], and Canada [30], we can say that family SES plays a key role in determining extra-curricular physical activity of students: the highest educational levels and the most remunerative work activities are directly related to the sporting practices of adolescents. Children with parents characterised by higher educational levels are encouraged more than others to practice constant and demanding extra-curricular sport. Zakarian and coll. [28] demonstrated that among minority and low-SES adolescents (high school population) in USA, the prevalence rate of vigorous exercise tends to decrease with age or when this kind of popuation is not required to participate in school physical education. McVeigh and coll. [29] found that South African children belonging to the highest SES quartile were highly physically active, watched less television and had greater lean tissue than children in lower quartiles. In Canada, among adolescents aged 12–20 years, individuals from low income families had a 30% higher probability of being inactive than those from high income families [30].

Families with high SES consider physical activity useful both in preventing chronic and degenerative diseases and for socialisation, essential for the correct physical and psychological development of adolescents.

SES is therefore clearly related to parents' educational levels, and parents' low educational levels and low remunerative work activities can negatively influence the participation of children in extra-curricular physical activity.

In addition, it must be considered the inadequate knowledge about benefits of physical activity, related to low socio-economic and cultural levels [31].

The presents study has some limitations. As far as concerns internal validity, we sampled randomly the participants, and used a validated questionnaire. Misclassification bias could arise if incorrect information on both exposure (SES status) and outcome (PA). The accuracy of self-reported physical activity of adolescents, should have been avoided, given the reliability of the tool we used, which had been validated in previous studies [17,18]. Moreover, we collected detailed information on potential confounding factors and took it into account in the analysis of possible effects

As far as external validity a potential selection bias could have been avoided, even if we used a cross-sectional type of study design. Finally, the precision of the estimates is comparable with that had been designed for.

Neverthless, to our knowledge this is the first attempt in Italy to evaluate the relationship between SES, based either on a family income index and parents' educational status, and adolescents' extra-curricular physical activity.

## Conclusion

In our study parents' physical activity is a good predictor of adolescents' extra-curriculum physical activity, accordingly to previous studies. In a recent review on correlates of physical activity of children and adolescents, Sallis and coll. [23] demonstrated that for children, among other factors, parental overweight status, physical activity preferences, intention to be active and program/facility access were statistically associated to physical activity. For adolescents, variables consistently associated with physical activity were perceived activity competence, intentions, but also sports conducted at community level, parent support, sibling physical activity, direct help from parents and opportunities to exercise. In that sense, social, cultural and economic deprivation have an effect on extra-curricular physical activity participation. A strong help in this task could derive from school. In fact, in Italy the vast majority of scholastic building and infrastructures belongs to municipalities and provinces, and are currently underused. A way to improve adolescents' participation in physical activity could be involving them in extra-curricular activity carried out within the schools. As an example, childhood obesity prevention might be achieved considering a variety of interventions targeting built environment, physical activity, and diet. As suggested by Dehghan and coll. (2005), some of these potential strategies for intervention in children can be implemented by targeting preschool institutions, schools or after-school care services as natural setting for influencing the diet and physical activity. In this sense, the outcomes from this study will be useful to school administrators in their bid to bridge the gap between those children who are the most and least deprived.

## Competing interests

The author(s) declared that they have no competing interests.

## Authors' contributions

All of th authors partecipated in the establishment of the research, and all read and approved the manuscript. GLT, DM and WR wrote the initial draft of the manuscript, which GLT edited.

## Pre-publication history

The pre-publication history for this paper can be accessed here:



## Figures and Tables

**Table 1:** Characteristics of student's parents.

	**Fathers (%)**	**Mothers (%)**
**Physical activity level**		
Intense (> twice/week)	277 (11.9)	219 (9.4)
Regular (twice/week)	356 (15.2)	376 (16.1)
Scarce (once/week)	454 (19.4)	392 (16.8)
Absent	1250 (53.5)	1346 (57.7)

**Educational level**		
Degree	377 (16.1)	403 (17.3)
High school	1101 (47.1)	1113 (47.7)
Junior high school	709 (30.3)	697 (29.9)
Primary school	151 (6.5)	122 (5.2)

**Work activity (2357/2392)**		
Manager/Professional	570 (24.2)	252 (10.6)
Office worker/Skilled worker	880 (37.3)	683 (28.7)
Non skilled worker/housewife	828 (35.1)	1430 (60)
Unemployed	34 (1.4)	14 (0.6)
Retired from work	45 (1.9)	3 (0.1)

**Table 2:** Results of the multiple logistic regression analysis on the association of independent variables on adolescents' "Extra-curricular physical activity"

**Model I**	
Dependent variable: "*extra-curricular physical activity"*	
**Independent variables**	***OR (95% CI)***

*Sex*	
Males (reference group)	1
Females	1.27 (1.04 – 1.56)*
*Father's educational level*	
Primary school	0.54 (0.37 – 0.80)**
Junior high school (reference group)	1
High school	1.26 (1.01 – 1.59)*
Degree	1.93 (1.36 – 2.72)**
*Father's physical activity*	
No (reference group)	1
Yes	1.58 (1.25 – 2.00)**
*Mother's physical activity*	
No (reference group)	1
Yes	1.31(1.03 – 1.66)*
*Physical activity is important in socialising*	
No (reference group)	1
Yes	2.46 (1.90 – 3.19)**
*Father's work activity*	
Non skilled worker (reference group)	1
Unemployed	0.36 (0.17 – 0.80)*
Manager/Professional	1.29 (1.01 – 1.67)*
Office worker/Skilled worker	1.11 (0.87 – 1.40)

Model II	
Dependent variable: "*extra-curricular physical activity"*	

**Independent variables**	***OR (95%CI)***

*Sex*	
Males (reference group)	1
Females	1.27 (1.04 – 1.55)*
*Father's educational level*	
Primary school	0.51 (0.35 – 0.73)**
Junior high school (reference group)	1
High school	1.17 (0.92 – 1.47)
Degree	1.44 (1.04 – 1.99)*
*Father's physical activity*	
No (reference group)	1
Yes	1.58 (1.25 – 2.01)**
*Mother's physical activity*	
No (reference group)	1
Yes	1.32 (1.07 – 1.68)*
*Physical activity is important in socialising*	
No (reference group)	1
Yes	2.34 (1.80 – 3.07)**
*Familial socio-economic index*	
Low and Middle-low (reference group)	1
Middle	1.23 (0.97 – 1.55)
High	1.63 (1.19 – 2.25)**
Very high	1.96 (1.43 – 2.69)**

* p < 0.05; ** p < 0.01
